# Exogenous NAD^+^ Postpones the D-Gal-Induced Senescence of Bone Marrow-Derived Mesenchymal Stem Cells via Sirt1 Signaling

**DOI:** 10.3390/antiox10020254

**Published:** 2021-02-07

**Authors:** Jie Wang, Lin Liu, Zhongjie Ding, Qing Luo, Yang Ju, Guanbin Song

**Affiliations:** 1 Key Laboratory of Biorheological Science and Technology, College of Bioengineering, Chongqing University, Chongqing 400030, China; wangjie518@cqu.edu.cn (J.W.); liulin@cqu.edu.cn (L.L.); dingzj@cqu.edu.cn (Z.D.); qing.luo@cqu.edu.cn (Q.L.); 2 Department of Mechanical Science and Engineering, Nagoya University, Nagoya 464-8603, Japan; ju@mech.nagoya-u.ac.jp

**Keywords:** mesenchymal stem cells, nicotinamide adenine dinucleotide, senescence, sirtuin 1 signaling

## Abstract

Cell senescence is accompanied by decreased nicotinamide adenine dinucleotide (NAD^+^) levels; however, whether exogenous NAD^+^ affects bone marrow-derived mesenchymal stem cells (BMSCs) senescence and the involved mechanisms is still unclear. Here, we find that exogenous NAD^+^ replenishment significantly postpones BMSC senescence induced by D-galactose (D-gal). It is also shown that exogenous NAD^+^ leads to increased intracellular NAD^+^ levels and reduced intracellular reactive oxygen species in senescent BMSCs here. Further investigation showed that exogenous NAD^+^ weakened BMSC senescence by increasing Sirtuin 1 (Sirt1) expression. Moreover, exogenous NAD^+^ reduced senescence-associated-β-galactosidase activity, and downregulated poly (ADP-ribose) polymerase 1 expression. In addition, the reduced expression of Sirt1 by small interfering RNA abolished the beneficial effects of exogenous NAD^+^ in terms of postponing BMSCs senescence induced by D-gal. Taken together, our results indicate that exogenous NAD^+^ could postpone D-gal-induced BMSC senescence through Sirt1 signaling, providing a potential method for obtaining high quality BMSCs to support their research and clinical application.

## 1. Introduction

Aging is a comprehensive manifestation of the decline of physiological functions and disorders of living organisms during their degradation period, and it is also a basic feature of life. Cell senescence is an irreversible phenomenon in which cellular functions and structures undergo degenerative changes over time and tend to die. Replicative senescence is a type of cellular senescence, which is specifically manifested as slow proliferation, growth stagnation, dryness, and loss of differentiation ability after normal cells undergo a limited number of divisions [1]. In general, Oncogene activation, sustained genome damage, hypoxia, shortened telomere length, and oxidative stress can cause replicative senescence in cells [2].

With the aging of the world’s population, aging-related Alzheimer’s disease, type II diabetes, and cancer have become important diseases that threaten human health. Thus, regenerative medicine and stem cell therapy have become viable strategies to treat aging-related diseases. Various adult stem cells maintain the homeostasis of the body through differentiation and participate in damaged tissue repair and regeneration [3]. Bone marrow-derived mesenchymal stem cells (BMSCs) are multipotent stem cells with a high self-renewal ability and multi-directional differentiation potential. Studies have found that in vitro cultured BMSCs can remain undifferentiated for a long time, are easily transferred, express foreign genes, and have low immunogenicity, leading to them showing great therapeutic potential for tissue engineering, genetic engineering, and stem cell transplantation. However, the content of BMSCs in the body is very small, so in vitro expansion is required to obtain a sufficient number of cells before clinical application. Unfortunately, in vitro cultured BMSCs are prone to replicative senescence [4]. Decreased cell function and stemness limit research and clinical applications. Therefore, it is particularly important to delay the aging of BMSCs.

The current methods of delaying cell senescence mainly focus on interfering with aging-related genes and protein expression or caloric restriction by adding drugs. Nicotinamide adenine dinucleotide (NAD^+^) is a common coenzyme of multiple metabolic enzymes in all living cells [5]. It can be used as a cofactor for key enzymes in glycolysis, the tricarboxylic acid cycle, and oxidative phosphorylation. It is involved in regulating various physiological and pathological activities, such as cell material metabolism, energy synthesis, and DNA damage repair [6]. Meanwhile, NAD^+^ is also a substrate of NAD^+^ hydrolases, such as Sirtuin 1 (Sirt1), CD38, and poly (ADP-ribose) polymerase 1 (PARP1), and it is involved in regulating both cell senescence and lifespan [7]. Sirt1 is a key mediator for postponing cell senescence, while PARP1 and CD38 play opposite roles [5,8]. Studies have found that NAD^+^ shows a downward trend during cell senescence [9], inhibiting NAD^+^ depleting enzyme activity or expression, or supplementing NAD^+^ and its precursors, showing great potential for the treatment of aging-related diseases caused by a decline in NAD^+^, suggesting that the remedy pathway for NAD^+^ may be key to maintaining cellular NAD^+^ levels and that NAD^+^ replacement therapy is suitable for targeting aging-related metabolism dysfunction [10]. Alano et al. demonstrated that supplementation with exogenous NAD^+^ eliminated ROS induced by amyloid β-peptide (Aβ) and reduced the oxidative damage, indicating that exogenous NAD^+^ protected neuronal DNA from Aβ-induced damage [11]. Other studies have shown that supplementation with exogenous NAD^+^ has effectively increased the expression of Sirt1, inhibited PARP1-mediated NAD^+^ depletion, and protected cells from damage induced by oxidative stress [7,12]. Similarly, studies by Zhu et al. and Liu et al. have shown that exogenous NAD^+^ increased intracellular NAD^+^ and Sirt1 activity, significantly reducing the H_2_O_2_-induced death of retinal pigment epithelial cells and H9c2 cells by upregulating autophagy and apoptosis [13,14]. 

Sirtuins and PARPs are two major enzymes in mammals that respond to NAD^+^ signals. Sirtuins are highly conserved NAD^+^-dependent deacetylases that sense changes in NAD^+^ levels and correspondingly transmit signals [5]. There are seven forms of Sirtuins in cells. Among them, Sirt1, as an anti-aging protein, reduces apoptosis and delays senescence by regulating apoptotic genes, oxidative stress, and energy metabolization [15]. Studies have shown that supplementation with exogenous NAD^+^ and its precursor substances activates Sirt1 and increases intracellular NAD^+^, which supports avoiding aging-related neurodegeneration, vascular sclerosis, and kidney disease [16,17,18,19]. Sirt1 knockout (KO) mice have shown an increased risk of diabetes [20]. When Sirt1 KO mice were fed a high-fat diet, they showed hepatic steatosis, insulin resistance, and other severe metabolic syndromes, suggesting that Sirt1 plays a vital role in regulating glucose and liver lipid homeostasis [21].

Although previous studies have shown that NAD^+^ plays an important role in various cellular senescence, there have been no reports on the effects of exogenous NAD^+^ on BMSC senescence. Therefore, this study focuses on exploring whether and how exogenous NAD^+^ protects BMSCs from senescence induced by D-gal.

## 2. Materials and Methods 

### 2.1. Cell Isolation and Culture

All experiments involving animals in this study were performed in accordance with the relevant ethical, national, and international standards, and this experiment was approved by the Chongqing Science and Technology Commission, Chongqing, China.

BMSCs were extracted by whole bone marrow adherence from healthy Sprague Dawley (SD) rats weighing about 100 g (Laboratory Animal Center, Chongqing Medical University, China). Briefly, the rat hind legs were completely isolated in a sterile environment, and, after removing the muscle tissue, the bone marrow cavity was gently blown with 10 mL of Dulbecco’s modified Eagle’s medium (DMEM, Gibco, MA, USA) supplemented with 10% fetal bovine serum (FBS, HyClone, logan, UT, USA). Additionally, 100 U/mL of penicillin and 100 μg/mL of streptomycin was added to the flasks. Then, the cell culture flasks were placed in an incubator in a 37 °C, 5% CO_2_, and 95% humidity environment for 24 h. After removing nonadherent cells in phosphate-buffered saline (PBS, Solarbio, Beijing, China), the media were changed every two days. After reaching 80–85% confluence, cells were digested with 0.25% trypsin containing 0.02% EDTA and then subcultured. The cells used in this study were passaged between 2 and 4 times.

### 2.2. Cell Viability Assay

Cell viability analysis was performed using the Cell Counting Kit-8 reagent (CCK-8, Biosharp, Wuhan, China). According to the instructions, cells were seeded in 96-well plates at a density of 5 × 10^3^ cells/well and incubated for 24 h. Then, the cells were treated with or without different concentrations of D-gal (Solarbio, Beijing, China) or NAD^+^ (Solarbio, Beijing, China) or knockdown Sirt1 by si-RNA and then incubated at 37 °C for another 48 h. After that, the culture media were removed, then mixing CCK-8 and the culture media in a volume ratio of 1: 10 which was then added to each well and incubated at 37 °C for 2 h. The absorbance was measured at a wavelength of 450 nm using a microplate reader in order to assess cell viability.

### 2.3. SA-β-Gal^+^ Activity Assay 

Histochemical staining of senescent BMSCs was performed using the SA-β-gal staining kit (Beyotime, Shanghai, China) according to the manufacturer’s instructions. Briefly, after induction, the culture media were removed, washed once with PBS, and the cells were fixed with a fixing solution for 30 min. Then, the samples were washed three times with PBS for 3 min each time and incubated with a new configuration for the staining working solution for 12–14 h without CO_2_. SA-β-gal-positive cells were blue. For quantification, Image J was used to count at least 300 cells in five random microscope fields (Leica, Wetzlar, Germany). The percentages of stained positive cells in each group were calculated, and the statistical results are shown in terms of how many blue cells were contained for every 100 cells.

### 2.4. Quantitative Real-Time Polymerase Chain Reaction

Total RNA was isolated from BMSCs using a high-purity RNA extraction kit (Biotake, Beijing, China). cDNA was synthesized using reverse transcription kit (Takara, Kusatsu, Japan). Quantitative real-time polymerase chain reaction (qRT-PCR) was performed using commercially available gene sequences (Tsingke, Beijing, China) by a CFX96™ real-time PCR detection system (Bio-Rad CFX Manager system, Hercules, CA, USA) according to the manufacturer’s instructions. The expression of the targeted gene was normalized to the expression of β-actin. The primer sequences were β-actin (XM_039089807.1): Forward, 5′-ACCGTCAGGTCACTATCG-3′, reverse, 5′-GGCATAGAGGTCTTTACGGATG-3′; sirt1 (NM_001372090): Forward, 5′-GGAACCTCTGCCTCATCTA-3′, reverse, 5′-CATACTCGCCACCTAACCT-3′; parp1 (NM_013063.2): Forward, 5′-TCACAGGGTCTGCGGATAGC-3′, reverse, 5′-GGTCAGGGGCGGTTTTGC-3′; cd38 (NP_037259.1): Forward, 5′-CTGCCAGGATAACTACCGACCT-3′, reverse, 5′-CTTTCCCGACAGTGTTGCTTCT-3′; p53 (NM_030989.3): Forward, 5′-CCAGGATGTTGCAGAGTTGTTAGA-3′, reverse, 5′-TTGAGAAGGGACGGAAGATGAC-3′.

### 2.5. Western Blotting

Total protein was extracted from BMSCs with a RIPA lysis buffer (Beyotime, Shanghai, China) supplemented with a 1% protease inhibitor (Solarbo, Beijing, China). Protein concentrations of the lysates were measured with a BCA protein assay kit (Beyotime, Shanghai, China). Then, equal amounts of each protein sample were separated with 10% SDS-PAGE gels and transferred onto polyvinylidene fluoride (PVDF) membranes (Millipore, MA, USA) by electroblotting. The blots were blocked for 1 h with 5% non-fat milk, then washed 3 times with TBS with 0.2% Tween-20 (TBST) for 10 min each time. The membranes were probed with rabbit anti-Sirt1 (1:1000, Cell Signaling Technology, Danvers, USA), rabbit anti-PARP1 (1:1000, ZENBIO, Chengdu, China), mouse anti-P53 (1:1000, Cell Signaling Technology, Danvers, MA, USA), rabbit anti-CD38 (1:1000, GeneTex, Alton, IL, USA), and mouse anti-β-actin (1:2000, ZSGB-BIO, Beijing, China) antibodies diluted in primary antibody diluents (Beyotime, Shanghai, China) overnight at 4 °C. Next, the membranes were incubated with anti-rabbit IgG (Bioss, Beijing, China) and anti-mouse IgG secondary antibodies (Bioss, Beijing, China) with 5% non-fat milk (Wandashan, Heilongjiang, China) for 1 h and then washed three times with TBST for 10 min each time. Proteins were visualized with an enhanced chemiluminescence kit (KeyGEN Biotech, Nanjing, China) using an image analyzer (Bio-Rad, Hercules, CA, USA) to quantify protein expression.

### 2.6. Detection of ROS

To determine changes in intracellular ROS, BMSCs were seeded in 6-well plates at a density of 2.5 × 10^4^ cells/well, with or without different concentrations of D-gal or NAD^+^ or knocked down Sirt1 by si-RNA, and then incubated at 37 °C for 48 h. According to the instructions for the ROS detection kit (Beyotime, Shanghai, China), after removing culture media, the cells were washed once with PBS, then added to a medium containing 10 μmol/L DCFH-DA (Beyotime, Shanghai, China) that was added to plates and then incubated for 30 min. The media were then removed, followed by washing three times with PBS. After trypsinization, the digestion was terminated with PBS containing 0.2% FBS and intracellular ROS levels were detected by flow cytometry (BD, New York, NY, USA). These results were analyzed using Flowjo-V10.

### 2.7. Quantification of Intracellular NAD^+^ and NAD^+^/NADH Ratio

The NAD^+^/NADH content colorimetric kit (Beyotime, Shanghai, China) was used to detect the intracellular NAD^+^ and NAD^+^/NADH ratio. The instructions were followed in order to make a standard curve. Then, the media in the 6-well plates were removed, followed by washing with PBS two or three times, the addition of 200 μL of a NAD^+^/NADH extract, and then gentle pipetting to promote cell lysis. To detect the intracellular NAD^+^/NADH ratio, 20 μL samples were pipetted into 96-well plates, then added to 90 μL of the currently prepared ethanol dehydrogenase working solution (88 μL reaction solution and 2 μL ethanol dehydrogenase), and then incubated at 37 °C for 10 min in the dark without CO_2_. Additionally, 10 μL of a chromogenic solution was added, then incubating for 30 min in dark. When detecting the NADH content, 50–100 μL samples were directly transferred into 96-well plates and incubated at 60 °C for 30 min before the detection of NADH. After centrifugation, 20 μL of the supernatant was pipetted into a 96-well plate. The remaining steps were the same as the NAD^+^/NADH detection steps, and the optical density (at 450 nm) was detected using a multi-well spectrophotometer (Bio-Rad, Hercules, CA, USA).

### 2.8. Small Interfering RNA Transfection

For the small interfering RNA (si-RNA) transfection experiments, BMSCs were seeded in 12-well plates at a density of 2 × 10^4^ cells/well. After inducing cell senescence by use of 10 g/L of D-gal for 48 h, according to the si-RNA instruction manual (Gima Gene, Shanghai, China), diluted si-RNA, si-RNA negative control, and lipofectamine 3000 (Invitrogen, MA, USA) with a FBS-free medium at a ratio of 2.5 μL/100 μL were added, respectively. Next, the si-RNA or negative control was mixed with lipofectamine 3000 and left to stand for 10–15 min, which was then added to an 800 μL medium with 10% FBS and mixed well. Finally, 1 mL of the above solution was added to each plate and incubated for another 48 h under a humidified atmosphere of 5% CO_2_ and 95% air at 37 °C. The si-Sirt1 primer sequence (XM_017601788.1) was si-RNA1-1: Forward, 5′-CCCUGUAAAGCUUUCAGAATT-3′, reverse, 5′-UUCUGAAAGCUUUACAGGGTT-3′; si-RNA1-2: Forward, 5′-CCAGUAGCACUAAUUCCAATT-3′, reverse, 5′-UUGGAAUUAGUGCUACUGGTT-3′; si-control: Forward, 5′-UUCUCCGAACGUGUCACGUTT-3′, reverse, 5′-ACGUGACACGUUCGGAGAATT-3′.

### 2.9. Statistical Analysis

All experiments in this study were repeated at least three times independently. All data are presented here as the mean ± standard deviation, and the results were statistically analyzed using Student’s t-test and analysis of variance. Differences were considered significant at *p* < 0.05.

## 3. Results

### 3.1. D-gal Induces BMSC Senescence

**Figure 1:**
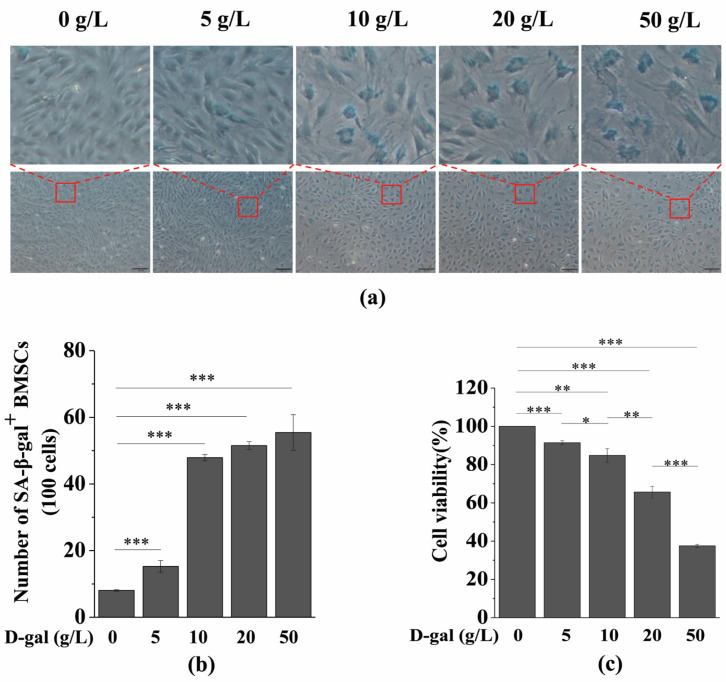
Effects of different concentrations of D-gal on BMSC senescence. BMSCs were induced by different concentrations of D-gal for 48 h. (**a**) Senescence-associated galactosidase staining was used to detect SA-β-gal activity (scale bar = 100 μm). The upper column is a partial enlarged view of the original image below. (**b**) Quantitative analysis of SA-β-gal-positive cells. The intensity of SA-β-gal staining was determined by means of the percentage of SA-β-gal^+^ cells (100 cells). (**c**) A CCK-8 assay was used to detect cell viability. The data are expressed as means ± standard deviation. *n* = 3, * *p* < 0.05; ** *p* < 0.01; *** *p* < 0.001. BMSCs: Bone marrow-derived mesenchymal stem cells; D-gal: D-galactose; SA-β-gal: Senescence-associated galactosidase staining; CCK-8: Cell Counting Kit-8.

The concentration of D-gal that induces cell senescence may vary depending on the cell type, so we constructed a model of D-gal-induced BMSC senescence. In this experiment, SA-β-gal staining and a cell viability assay were performed to determine the optimal concentration of D-gal to induce BMSC senescence. As shown in Figure 1a,b, only a few BMSCs in the control group were SA-β-gal^+^ cells. However, the number of SA-β-gal^+^ cells significantly increased with an increasing D-gal concentration. Meanwhile, the cell viability also decreased significantly with an increasing D-gal concentration (Figure 1c). Considering these results, the experimental group with a relatively high number of SA-β-gal^+^ cells and a relatively small decrease in cell viability meets the requirements. Therefore, 10 g/L of D-gal was used to construct the aging model for BMSCs. 

### 3.2. Exogenous NAD^+^ Postpones BMSC Senescence Induced by D-Gal

**Figure 2:**
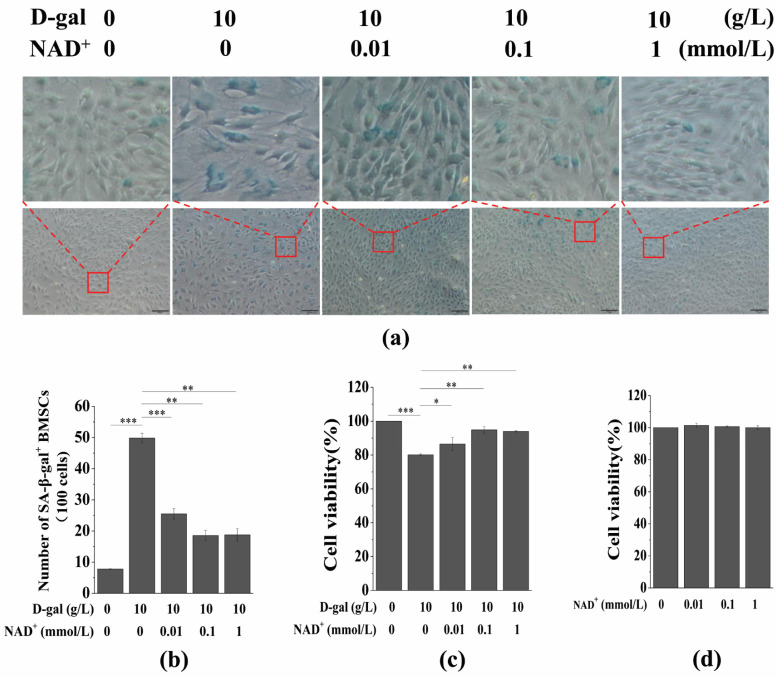
Effects of exogenous NAD^+^ on senescent BMSCs. BMSCs were induced by 10 g/L of D-gal for 48 h and then were treated with different concentrations of exogenous NAD^+^ for another 48 h. (**a**) Senescence-associated galactosidase staining was used to detect SA-β-gal activity (scale bar = 100 μm). The upper column is a partial enlarged view of the original image below. (**b**) Quantitative analysis of SA-β-gal-positive cells in (**a**). The intensity of SA-β-gal staining was determined by means of the percentage of SA-β-gal^+^ cells (100 cells). (**c**) A CCK-8 assay was used to detect cell viability. (**d**) Normal BMSCs were treated by different concentrations of exogenous NAD^+^ for 48 h, and CCK-8 was used to detect the cell viability. Different concentrations of exogenous NAD^+^ had no significant effect on the cell viability of normal BMSCs. The data are expressed as means ± standard deviation. *n* = 3, * *p* < 0.05; ** *p* < 0.01; *** *p* < 0.001. BMSCs: Bone marrow-derived mesenchymal stem cells; D-gal: D-galactose; NAD^+^: Nicotinamide adenine dinucleotide; SA-β-gal: Senescence-associated galactosidase staining.

To investigate the effect of exogenous NAD^+^ on senescent BMSCs, the cells were treated with different concentrations of NAD^+^ (0.01, 0.1, and 1 mmol/L), and then SA-β-gal staining and a CCK-8 assay were used to detect SA-β-gal activity and cell viability, respectively. The results showed that the number of SA-β-gal^+^ cells decreased significantly with an increasing NAD^+^ concentration when compared the results for the D-gal-induced group (Figure 2a,b). Moreover, the cell viability increased significantly with an increasing NAD^+^ concentration (Figure 2c). In addition, the CCK-8 assay showed that different concentrations of NAD^+^ had no obvious toxic effects or side effects on normal BMSCs (Figure 2c). These results suggest that exogenous NAD^+^ increases the cell viability of senescent BMSCs and protects them from D-gal-induced senescence.

### 3.3. Exogenous NAD^+^ Increases the Intracellular NAD^+^ Content and NAD^+^/NADH Ratio 

**Figure 3:**
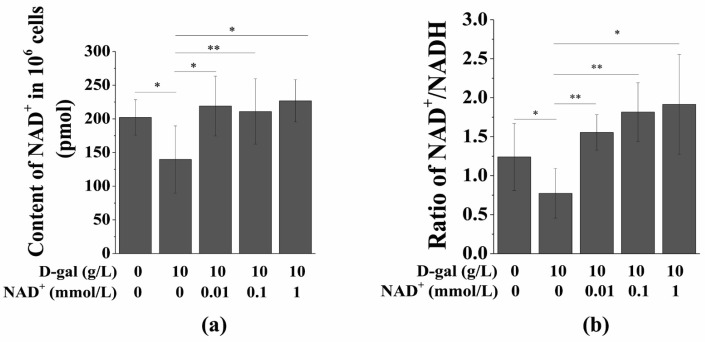
Exogenous NAD^+^ restores NAD^+^ levels in senescent BMSCs. BMSCs were induced by 10 g/L of D-gal for 48 h and then were treated by different concentrations of exogenous NAD^+^ for another 48 h. (**a**) A NAD^+^/NADH content colorimetric kit was used to detect the changes of NAD^+^ levels in senescent BMSCs. (**b**) Changes of NAD^+^/NADH in senescent BMSCs determined by the NAD^+^/NADH content colorimetric kit. The data are expressed as means ± standard deviation. *n* = 3; * *p* < 0.05; ** *p* < 0.01. BMSCs: Bone marrow-derived mesenchymal stem cells; D-gal: D-galactose; NAD^+^: Nicotinamide adenine dinucleotide.

Numerous studies have shown that the NAD^+^ content in the body or cells decreases significantly with aging, and intracellular NAD^+^ depletion leads to cell senescence and even death [20,21]. Considering the protective effect of exogenous NAD^+^ on senescent BMSCs, we speculated that exogenous NAD^+^ may protect cells from D-gal-induced senescence by increasing the amount of intracellular NAD^+^. To test this hypothesis, we examined changes of intracellular NAD^+^ and the NAD^+^/NADH ratio in senescent BMSCs treated with or without exogenous NAD^+^. As shown in Figure 3, the intracellular NAD^+^ and NAD^+^/NADH significantly decreased in the D-gal-treated group when compared to the control group. However, after exposure to NAD^+^, intracellular NAD^+^ and NAD^+^/NADH in BMSCs were significantly increased when compared with the D-gal group that did not feature NAD^+^ treatment (Figure 3a,b). These results indicate that the protective effect of exogenous NAD^+^ on senescent BMSCs may be achieved by restoring intracellular NAD^+^.

### 3.4. Exogenous NAD^+^ Inhibits Intracellular ROS Generation in Senescent BMSCs

**Figure 4:**
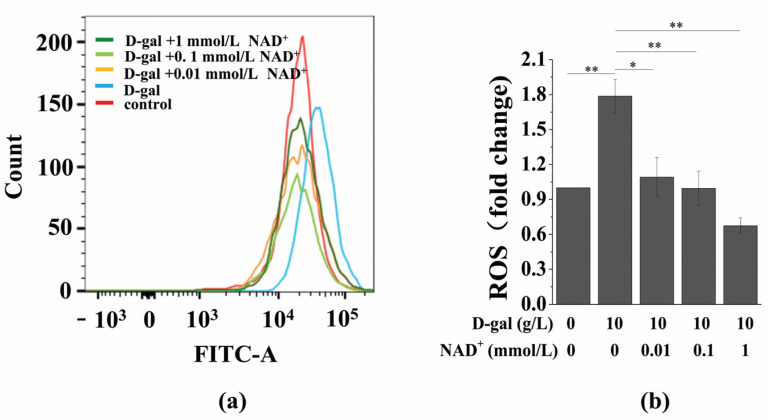
Exogenous NAD^+^ downregulates intracellular ROS in senescent BMSCs. BMSCs were induced by 10 g/L of D-gal for 48 h and were treated by different concentrations of exogenous NAD^+^ for another 48 h. (**a**) Detection of intracellular ROS by flow cytometry. (**b**) Quantitative analysis of intracellular ROS. The data are expressed as means ± standard deviation. *n* = 3; * *p* < 0.05; ** *p* < 0.01. BMSCs: Bone marrow-derived mesenchymal stem cells; D-gal: D-galactose; NAD^+^: Nicotinamide adenine dinucleotide; ROS: Reactive oxygen species.

Oxidative stress is an important cause of cell senescence. High concentrations of ROS cause oxidative damage to various organelles and induce cell senescence or death [9,22,23,24]. It is known that D-gal generates a large amount of ROS under the action of galactosidase, which causes cell dysfunction and promotes cell senescence. Therefore, we tested the effect of exogenous NAD^+^ on intracellular ROS in senescent BMSCs. The results showed that D-gal significantly upregulated intracellular ROS, while exogenous NAD^+^ reversed this phenomenon (Figure 4).

### 3.5. Exogenous NAD^+^ Regulates the Expression of Sirt1 and PARP1 in Senescent BMSCs

**Figure 5:**
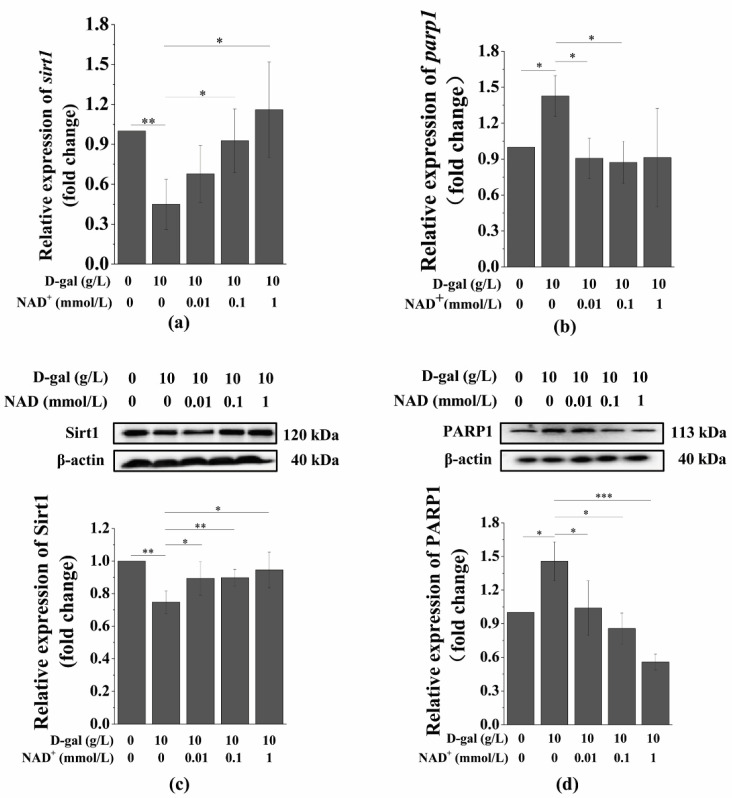
Exogenous NAD^+^ regulates the expression of Sirt1 and PARP1 in senescent BMSCs. BMSCs were induced by 10 g/L of D-gal for 48 h and were treated by different concentration of exogenous NAD^+^ for another 48 h. Quantitative real-time polymerase chain reaction (qRT-PCR) was used to detect the expression of *sirt1* (**a**) and *parp1* (**b**) in senescent BMSCs. Western blotting (WB) was used to detect the expression of Sirt1 (**c**) and PARP1 (**d**) in senescent BMSCs, and the values below the bands are their quantitative results. The data are expressed as means ± standard deviation. *n* = 3; * *p* < 0.05; ** *p* < 0.01; *** *p* < 0.001. BMSCs: Bone marrow-derived mesenchymal stem cells; D-gal: D-galactose; NAD^+^: Nicotinamide adenine dinucleotide; Sirt1: Sirtuin 1; PARP1: Poly (ADP-ribose) polymerase 1.

Studies have shown that Sirtuins and PARPs are two major enzymes in mammals that respond to NAD^+^ signals. As reported before, Sirt1 senses changes in intracellular NAD^+^ and transmits corresponding signals through NAD^+^-dependent protein deacetylation [21]. We have found that intracellular NAD^+^ significantly increased with exogenous NAD^+^ in senescent BMSCs. To determine whether Sirt1 was involved in the protection of senescent BMSCs by exogenous NAD^+^, we first examined the expression of the Sirt1 protein in senescent BMSCs with or without exogenous NAD^+^. It can be seen from the results that the expression of Sirt1 in D-gal-induced BMSCs were significantly downregulated; however, exogenous NAD^+^ restored the expression of Sirt1 at both mRNA (Figure 5a) and protein (Figure 5c) levels in senescent BMSCs. It is well known that P53 is a downstream target of the Sirt1 anti-aging pathway, and studies have shown that mouse P53 KO delayed the senescence of ovaries. We found that exogenous NAD^+^ downregulates D-gal-induced *p53* (Figure S1a) and its protein (Figure S1b) expression. 

Intracellular NAD^+^ depletion and excess intracellular ROS upregulate the expression of PARP1. We have found that exogenous NAD^+^ increased intracellular NAD^+^ and inhibited intracellular ROS generation in senescent BMSCs; however, the effect of exogenous NAD^+^ on PARP1 in senescent BMSCs was unknown. Therefore, we further investigated whether exogenous NAD^+^ affects the expression of PARP1. The results showed that PARP1 in senescent BMSCs was significantly higher than that in the control group, while exogenous NAD^+^ significantly downregulated the expression of PARP1 at both mRNA (Figure 5b) and protein (Figure 5d) levels in senescent BMSCs. Furthermore, recent studies have suggested that CD38 is also related to senescence induced by D-gal and competes with Sirt1 and PARP1 for intracellular NAD^+^. Camacho-Pereira et al. have shown that reduced intracellular NAD^+^ is associated with increased CD38 expression in senescent mice [9]. Our results showed that the effect of exogenous NAD^+^ on CD38 in senescent BMSCs is consistent with the finding of PARP1; the expression of CD 38 was significantly downregulated both at mRNA (Figure S1b) and at protein (Figure S1d) levels. These results indicate that exogenous NAD^+^ may protect BMSCs from D-gal-induced senescence by regulating Sirt1 and PARP1 expression.

### 3.6. Sirt1 Knockdown Suppresses the Exogenous NAD^+^-Affected Expression of Sirt1 and PARP1 in Senescent BMSCs

**Figure 6:**
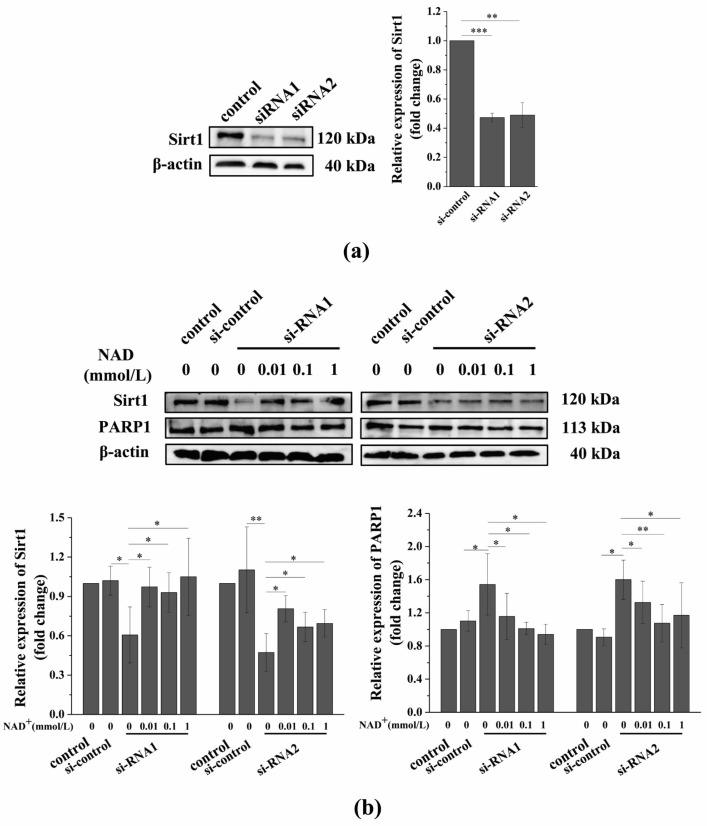
Sirt1 knockdown suppresses exogenous NAD^+^-affected expression of Sirt1 and PARP1 in senescent BMSCs. (**a**) BMSCs were treated by two different siRNA sequences for 48 h and WB was used to detect the expression of Sirt1, and values next to the bands show the quantitative results. (**b**) After the knockdown of Sirt1 by si-RNA1 or si-RNA2 sequences for 48 h, D-gal-induced senescent BMSCs were treated with different concentrations of NAD^+^ for another 48 h. WB was used to detect the expressions of Sirt1 and PARP1, and values below the bands are their quantitative results. The data are expressed as means ± standard deviation. *n* ≥ 3; * *p* < 0.05; ** *p* < 0.01; *** *p* < 0.001. BMSCs: Bone marrow-derived mesenchymal stem cells; D-gal: D-galactose; NAD^+^: Nicotinamide adenine dinucleotide; Sirt1: Sirtuin 1; PARP1: Poly (ADP-ribose) polymerase 1.

Our previous results that suggest that exogenous NAD^+^ postpones the D-gal-induced senescence of BMSCs may be related to the expression of Sirt1 and PARP1. However, Sirt1 and PARP1 not only compete with each other for intracellular NAD^+^, but also have dense crosstalk through cross-modification and transcriptional regulation. As a result, Sirt1 and PARP1 might be able to cancel each other’s regulation of cell survival [25]. Our aforementioned experimental results show that exogenous NAD^+^ significantly upregulated the expression of Sirt1 and inhibited the expression of PARP1 in senescent BMSCs. Thus, we further investigated the role of Sirt1 in postponing D-gal-induced BMSC senescence with exogenous NAD^+^ by the knockdown of Sirt1 with si-RNA. These findings revealed that Sirt1 expression was significantly downregulated after the knockdown of Sirt1 (Figure 6a,b). In contrast, the expressions of PARP1 (Figure 6b), P53 (Figure S2a), and CD38 (Figure S2b) in senescent BMSCs were significantly upregulated. Fortunately, exogenous NAD^+^ reversed the expression changes of these molecules in senescent BMSCs featuring Sirt1 knockdown, although the expressions of these proteins were not completely restored when compared to normal BMSCs (Figure 6). However, the changes of these protein expressions and the concentration of exogenous NAD^+^ did not show a specific pattern in this study.

### 3.7. Sirt1 Knockdown Represses Exogenous NAD^+^-Inhibited ROS Generation and D-Gal-Induced BMSC Senescence

**Figure 7:**
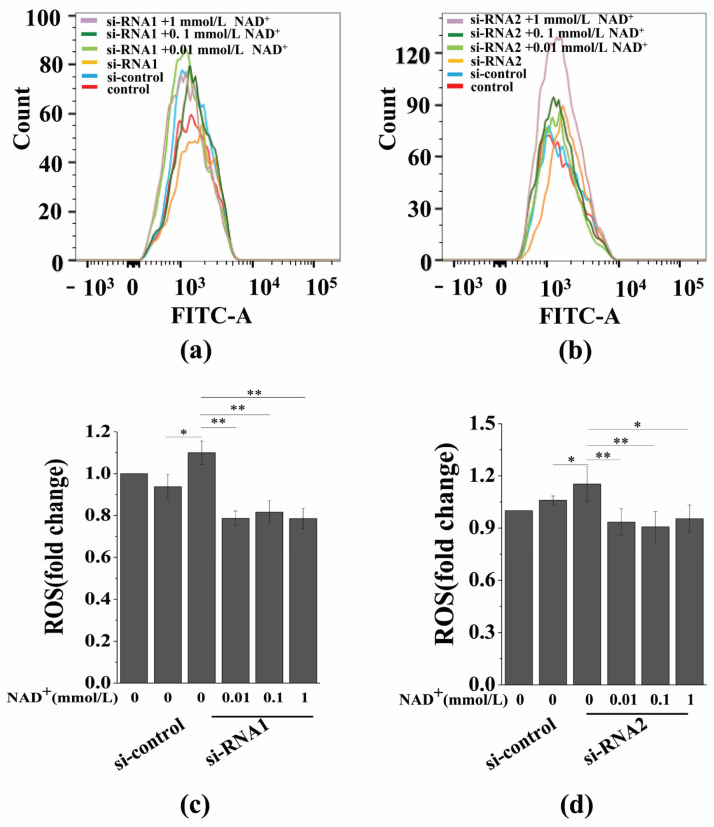
Sirt1 knockdown represses exogenous NAD^+^-inhibited intracellular ROS generation. (**a**,**b**) After the knockdown of Sirt1 by two different si-RNA sequences for 48 h, D-gal-induced senescent BMSCs were treated with different concentrations of NAD^+^ for another 48 h. The detection of intracellular ROS was carried out by flow cytometry. (**c**,**d**) Quantitative analysis of intracellular ROS. The data are expressed as means ± standard deviation; *n* = 3; * *p* < 0.05; ** *p* < 0.01. BMSCs: Bone marrow-derived mesenchymal stem cells; D-gal: D-galactose; NAD^+^: Nicotinamide adenine dinucleotide; ROS: Reactive oxygen species.

**Figure 8:**
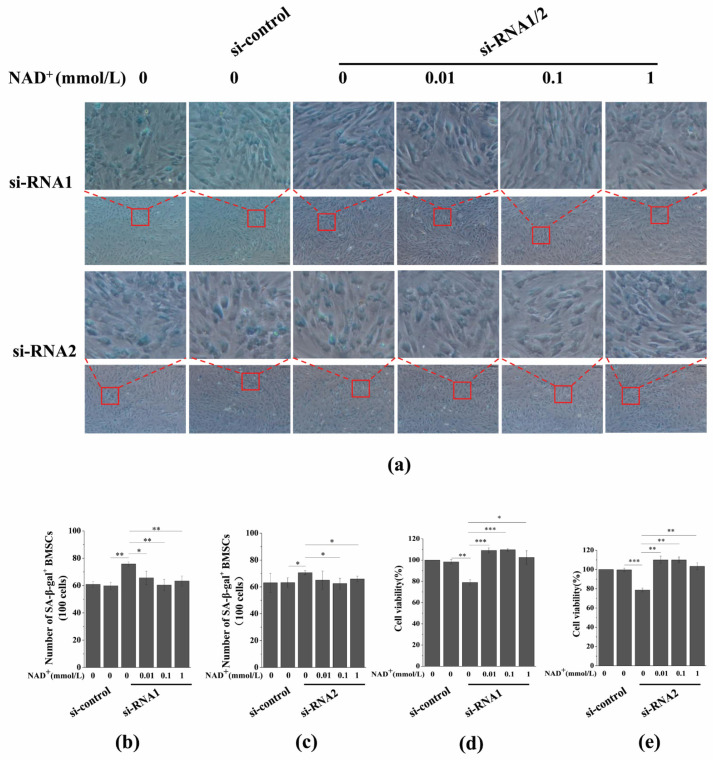
Sirt1 knockdown represses exogenous NAD^+^-inhibited D-gal-induced BMSC senescence. After the knockdown of Sirt1 by two different si-RNA sequences for 48 h, D-gal-induced senescent BMSCs were treated with different concentrations of NAD^+^ for another 48 h. (**a**) A SA-β-gal assay was used to detect the SA-β-gal activity (scale bar = 100 μm). The upper column is a partial enlarged view of the original image below. (**b**,**c**) Quantitative analysis of SA-β-gal-positive cells in (**a**). The intensity of SA-β-gal staining was determined by means of the percentage of SA-β-gal^+^ cells (100 cells). The data are expressed as means ± standard deviation. (**d**,**e**) A CCK-8 assay was used to detect cell viability. The data are expressed as means ± standard deviation. *n* = 3; * *p* < 0.05; ** *p* < 0.01; *** *p* < 0.001. BMSCs: Bone marrow-derived mesenchymal stem cells; D-gal: D-galactose; SA-β-gal staining: Senescence-associated galactosidase staining; NAD^+^: Nicotinamide adenine dinucleotide; Sirt1: Sirtuin 1; PARP1: Poly (ADP-ribose) polymerase 1.

To further evaluate the role of Sirt1 in mediating NAD^+^-affected BMSC senescence induced by D-gal, we determined the effect of exogenous NAD^+^ on intracellular ROS generation after Sirt1 knockdown in senescent BMSCs. Our results showed that intracellular ROS generation significantly increased by knockdown Sirt1, and, importantly, Sirt1 knockdown by si-RNA1 (Figure 7a,c) or si-RNA2 (Figure 7b,d) reduced the inhibitory effect of exogenous NAD^+^ on ROS generation in senescent BMSCs. In addition, SA-β-gal staining and the CCK-8 assay showed that the knockdown of Sirt1 could significantly promote the D-gal-induced senescence of BMSCs, while reducing the protective effect of exogenous NAD^+^ on senescent BMSCs (Figure 8a–c), and the viability of BMSCs was significantly reduced (Figure 8d,e). Regrettably, Sirt1 knockdown attenuated exogenous NAD^+^-inhibited ROS generation and BMSC senescence induced by D-gal. Collectively, these results demonstrated that exogenous NAD^+^ regulated D-gal-induced BMSC senescence through Sirt1 signaling.

As an activator of Sirt1, resveratrol (Res) is a natural anti-aging agent and has shown good anti-cancer and anti-aging effects [26]. In order to compare the difference between Res and NAD^+^-delayed BMSCs, we also evaluated the effects of Res and NAD^+^ on senescent BMSCs. The results suggested that both of them can significantly delay D-gal-induced BMSC senescence, and that there is no significant difference between their efficacy (Figure S3).

## 4. Discussion

At present, strategies for postponing the senescence of BMSCs are still not sufficient. NAD^+^ has been shown to play an important role in cellular senescence, but the role and mechanisms of exogenous NAD^+^ with senescent BMSCs is still unclear. In the present study, we have demonstrated that exogenous NAD^+^ postpones D-gal-induced BMSC senescence and increases intracellular NAD^+^ in senescent BMSCs. The knockdown of Sirt1 promoted the D-gal-induced senescence of BMSCs and weakened the effect of exogenous NAD^+^ in senescent BMSCs. Our results suggested that Sirt1 plays a key role in mediating exogenous NAD^+^-postponed BMSC senescence induced by D-gal.

The self-renewal and differentiation capabilities of BMSCs have made them widely used in scientific research and regenerative medicine, but more and more evidence has shown that the senescence of BMSCs affects their application in various aspects. The role of NAD^+^ in individual senescence, inflammation, and energy metabolization has been thoroughly studied [27,28]. To our knowledge, this is the first study to demonstrate that exogenous NAD^+^ protects BMSCs from D-gal-induced senescence. Nevertheless, other studies reported that oxidative stress and oncogenes induce cell senescence in different mechanisms from D-gal [29,30], whether exogenous NAD^+^ can protect senescent BMSCs induced by other factors through the same mechanism remains to be further studied. This research extends previous findings that have shown that exogenous NAD^+^ supplementation has significant therapeutic effects on cardiac hypertrophy [31], oxidative damage to retinal epithelial cells and H9c2 cardiomyocytes [13,14], and senescent muscle stem cells [15]. In addition, exogenous NAD^+^ may have the same effect on other mesenchymal stem cells (MSCs) with similar cellular functions and properties to BMSC, such as dental pulp stem cells [32]. This will greatly improve the application of MSCs cultured in vitro in regenerative medicine. Interestingly, other studies have found that NAD^+^ depletion may have beneficial effects for the treatment of tumors. Cancer cells obtain high energy by upregulating energy metabolization, providing conditions for rapid proliferation [33]. At present, aerobic glycolysis has become the target of anticancer therapy. NAD^+^ is a key coenzyme of aerobic glycolytic enzymes [34], and reducing NAD^+^ levels will slow the rate of aerobic glycolytics [35]. Studies have shown that NAD^+^ levels are much higher in cancer cells compared to normal cells. By inhibiting NAMPT, a key enzyme in NAD^+^ biosynthesis, reducing NAD^+^ levels can significantly inhibit tumor growth and improve patient survival [10,36]. NAD^+^ regulates DNA damage repair by mediating PARP1 activity. Decreased NAD^+^ levels inhibit the DNA repair process and prevent cancer cell proliferation [33]. These results indicate that the significance of changes in the regulation of NAD^+^ levels varies for different cell types. In addition, the mechanism by which exogenous NAD^+^ affects intracellular NAD^+^ is still not fully understood. A study by Pillai et al. showed that NAD^+^ enters and exits myocardial cells through the Cx43 half channel on the membrane [37]. However, it remains to be explored whether NAD^+^ can also enter and exit BMSCs or other cell types through this channel. 

It has been found that resveratrol, as an activator of Sirt1, has obvious antioxidant and anti-aging effects, and significantly inhibits osteoporosis and endothelial cell senescence in rats. Currently, there is also research that has reported that resveratrol has delayed senescence in human BMSCs and mouse ovarian stem cells [38,39]. Besides, resveratrol activates the osteogenic differentiation of BMSCs and reduces osteoclast function by reducing oxidative stress [40]. In the present study, our results have demonstrated that resveratrol significantly postpones D-gal-induced BMSC senescence. Although there is no significant difference in the effects of resveratrol and NAD^+^ in postponing D-gal-induced BMSC senescence, due to the expensive price of resveratrol, while NAD^+^ is cheaper and easier to obtain, our results suggested that NAD^+^ could be widely used as a new and cheap anti-aging agent for BMSCs.

Sirtuins have been shown to regulate a variety of cellular pathways and physiological processes [21]. Among them, as an anti-aging protein, Sirt1 reduces apoptosis and the senescence of cells by regulating the expression of apoptotic genes, oxidative stress, and energy metabolization. Studies have shown that the expression of Sirt1 has a beneficial effect in terms of weakening osteoporosis in rats [41], the formation of fear memory in mice [42], and delaying the senescence of hepatocellular carcinoma (HCC) cells [43]. Here, we revealed the protective effect of exogenous NAD^+^ on senescent BMSCs. Interestingly, this beneficial effect is related to the upregulation of Sirt1 expression. The data from the Sirt1 knockdown experiments confirmed this theory; therefore, the upregulation of Sirt1 expression induced by exogenous NAD^+^ plays a key role in postponing D-gal-induced BMSC senescence. The research by Yuan et al. showed that conditioned media from MSCs upregulate the expression of Sirt1, enhance mitochondrial biogenesis, and protect endothelial cells from high-concentration glucose-induced oxidative damage. Zhao et al. found that upregulated Sirt1 effectively delays the senescence of HCC cells when induced by oxidative damage [43]. These studies suggest that Sirt1 expression is closely related to oxidative damage. It is interesting that the results of our study show that exogenous NAD^+^ significantly inhibits intracellular ROS generation, and that Sirt1 knockdown represses exogenous NAD^+^-inhibited intracellular ROS generation. Based on these results, it is obvious that ROS is an important factor for regulating BMSC senescence [44]. This study has revealed that exogenous NAD^+^ may attenuate ROS-induced oxidative damage in senescent BMSCs by upregulating Sirt1 expression, confirming once again the crucial role of Sirt1 in postponing D-gal-induced cell senescence.

## 5. Conclusions

Overall, this study confirms that exogenous NAD^+^ postpones D-gal-induced BMSC senescence through Sirt1 signaling. Our findings provide a theoretical basis and methodological reference for the widespread application of NAD^+^ in the field of anti-aging and the acquisition of high-quality BMSCs in vitro and their better clinical application.

## Data Availability

All data is contained within the article.

## Supplementary Materials

The following are available online at https://www.mdpi.com/2076-3921/10/2/254/s1, Figure S1: Exogenous NAD^+^ downregulates the expression of CD38 and P53 in senescent BMSCs. Figure S2: Sirt1 knockdown suppresses the exogenous NAD^+^-affected expression of CD38 and P53 in senescent BMSCs. Figure S3: Effects of exogenous NAD^+^ and resveratrol on aging BMSCs.
