# Supplementary material for: Exogenous NAD+ Postpones the D-Gal-Induced Senescence of Bone Marrow-Derived Mesenchymal Stem Cells via Sirt1 Signaling
Source: Antioxidants (Basel). 2021 Feb 7;10(2):254. doi: 10.3390/antiox10020254 (PMC7915830; doi:10.3390/antiox10020254)
Supplement: Supplementary file 1 [file antioxidants-10-00254-s001.pdf]

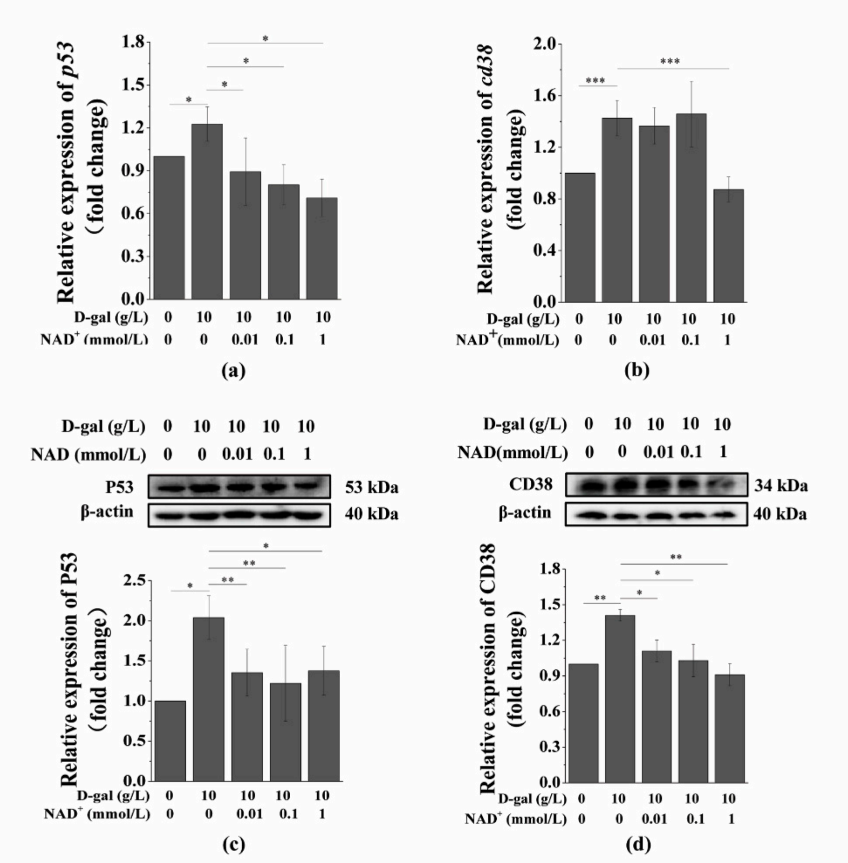


Figure S1: Exogenous NAD+ downregulates the expression of CD38 and P53 in senescent BMSCs. BMSCs were induced by 10 g/L of D-gal for 48 h and were treated by different concentrations of exogenous NAD+ for another 48 h. Quantitative real-time polymerase chain reaction (qRT-PCR) was used to detect the expression of *sirt1* (a) and *parp1* (b) in senescent BMSCs. Western blotting (WB) was used to detect the expression of Sirt1 (c) and PARP1 (d) in senescent BMSCs, and values next to the bands show the quantitative results. The data are expressed as means ± standard deviation. n = 3; *$p < 0.05$; **$p < 0.01$. BMSCs: Bone marrow-derived mesenchymal stem cells; D-gal: D-galactose; NAD+: Nicotinamide adenine dinucleotide.

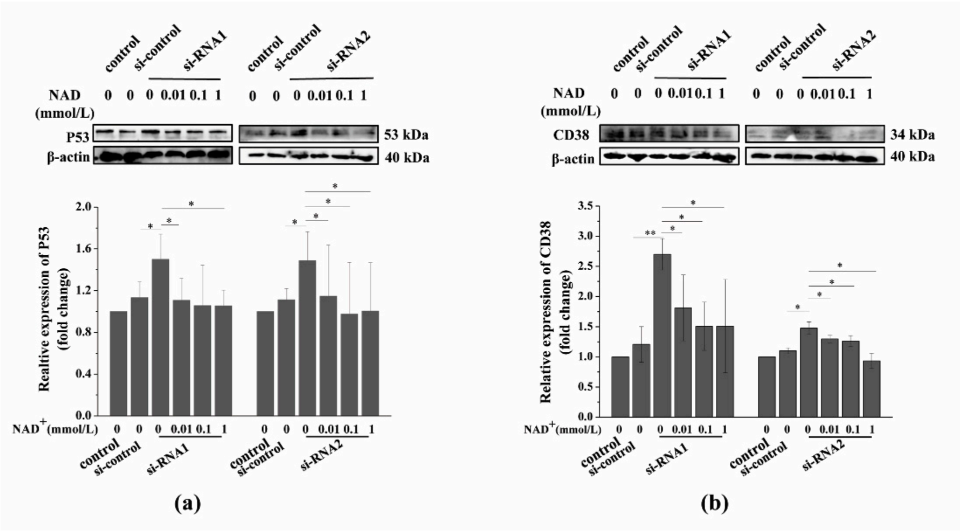


Figure S2: Sirt1 knockdown suppresses the exogenous NAD+-affected expression of CD38 and P53 in senescent BMSCs. The senescent BMSCs were treated by two different si-RNA sequences for 48 h and were treated with different concentrations of NAD+ for another 48 h. WB was used to detect the expressions of P53 (a) and CD38 (b), and values next to the bands show the quantitative results. The data are expressed as means ± standard deviation. $n \geq 3$; **p* $<$0.05; ***p* $<$0.01. BMSCs: Bone marrow-derived mesenchymal stem cells; D-gal: D-galactose; NAD+: Nicotinamide adenine dinucleotide.

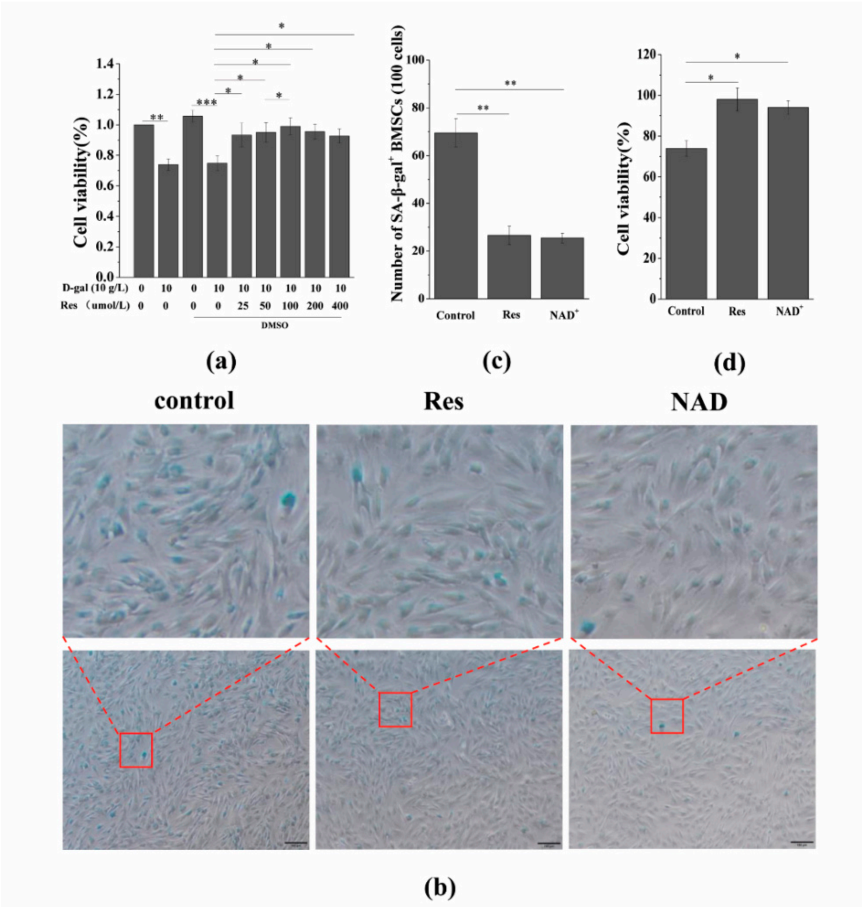


Figure S3: Effects of exogenous NAD$^+$ and resveratrol on aging BMSCs. BMSCs were induced by 10 g/L of D-gal for 48 h and were treated by different concentrations of exogenous NAD$^+$ or Res for another 48 h. (a) CCK-8 was used to determine the effects of different concentrations of Res on the viability of senescent BMSCs. In the experiment, all Res treatment group contained DMSO, and the highest DMSO concentration was 0.4%. (b) Senescence-associated galactosidase staining was used to detect SA-β-gal activity (scale bar = 100 μm). The upper column is a partial enlarged view of the original image below. (c) Quantitative analysis of SA-β-gal-positive cells in (b). The intensity of SA-β-gal staining was determined by means of the percentage of SA-β-gal$^+$ cells (100 cells). (d) A CCK-8 assay was used to determine the effects of Res and NAD$^+$ on the viability of senescent BMSCs. The data are expressed as means ± standard deviation. n = 3; *$p < 0.05$; **$p < 0.01$; ***$p < 0.001$. BMSCs: Bone marrow-derived mesenchymal stem cells; D-gal: D-galactose; NAD$^+$: Nicotinamide adenine dinucleotide; SA-β-gal: Senescence-associated galactosidase staining; Res: Resveratrol.
